# Helical Defects in MicroRNA Influence Protein Binding by TAR RNA Binding Protein

**DOI:** 10.1371/journal.pone.0116749

**Published:** 2015-01-21

**Authors:** Roderico Acevedo, Nichole Orench-Rivera, Kaycee A. Quarles, Scott A. Showalter

**Affiliations:** Department of Chemistry and Center for RNA Molecular Biology, The Pennsylvania State University, University Park, Pennsylvania, United States of America; George Mason University, UNITED STATES

## Abstract

**Background:**

MicroRNAs (miRNAs) are critical post-transcriptional regulators of gene expression. Their precursors have a globally A-form helical geometry, which prevents most proteins from identifying their nucleotide sequence. This suggests the hypothesis that local structural features (e.g., bulges, internal loops) play a central role in specific double-stranded RNA (dsRNA) selection from cellular RNA pools by dsRNA binding domain (dsRBD) containing proteins. Furthermore, the processing enzymes in the miRNA maturation pathway require tandem-dsRBD cofactor proteins for optimal function, suggesting that dsRBDs play a key role in the molecular mechanism for precise positioning of the RNA within these multi-protein complexes. Here, we focus on the tandem-dsRBDs of TRBP, which have been shown to bind dsRNA tightly.

**Methodology/Principal Findings:**

We present a combination of dsRNA binding assays demonstrating that TRBP binds dsRNA in an RNA-length dependent manner. Moreover, circular dichroism data shows that the number of dsRBD moieties bound to RNA at saturation is different for a tandem-dsRBD construct than for constructs with only one dsRBD per polypeptide, revealing another reason for the selective pressure to maintain multiple domains within a polypeptide chain. Finally, we show that helical defects in precursor miRNA alter the apparent dsRNA size, demonstrating that imperfections in RNA structure influence the strength of TRBP binding.

**Conclusion/Significance:**

We conclude that TRBP is responsible for recognizing structural imperfections in miRNA precursors, in the sense that TRBP is unable to bind imperfections efficiently and thus is positioned around them. We propose that once positioned around structural defects, TRBP assists Dicer and the rest of the RNA-induced silencing complex (RISC) in providing efficient and homogenous conversion of substrate precursor miRNA into mature miRNA downstream.

## Introduction

Multicellular organisms employ complex gene regulatory programs that often include post-transcriptional controls in order to establish gene-appropriate expression levels. Among the most studied mechanisms relevant to higher eukaryotes is post-transcriptional gene silencing carried out in RNA interference (RNAi). MicroRNAs (miRNAs) and small interfering RNAs (siRNA) are single-stranded RNAs that bind to complementary sequences found in the 3′-untranslated region of mRNA and down-regulate protein expression.[1,2] Both miRNA and siRNA are produced initially as double-stranded RNA (dsRNA) precursors: primary miRNA (∼44 imperfect base pairs) have a generally conserved hairpin-loop overall structure while siRNA precursors are long dsRNA (∼100’s base pairs).[3] Biochemical experiments show that in humans more than 60% of genes are regulated by miRNAs,[4] and bioinformatics analysis predicts that over 90% of translated genes could be regulated in this manner.[5] MiRNAs are critical for processes such as apoptosis, cell proliferation, and cell development;[6,7] therefore, depletion or mutation of miRNAs has been linked to multiple diseases.[8–10] For instance, alterations in the expression of miR-16-1 can lead to many types of cancers and are especially prevalent in chronic lymphocytic leukemia.[11–13] Establishing the molecular mechanisms governing the functions of processing proteins involved in the RNAi pathway, especially the cofactors that are required to process miRNAs to maturation, will lead to a deeper understanding of the roles that miRNA play in the etiology of multiple diseases.

**Figure 1:**
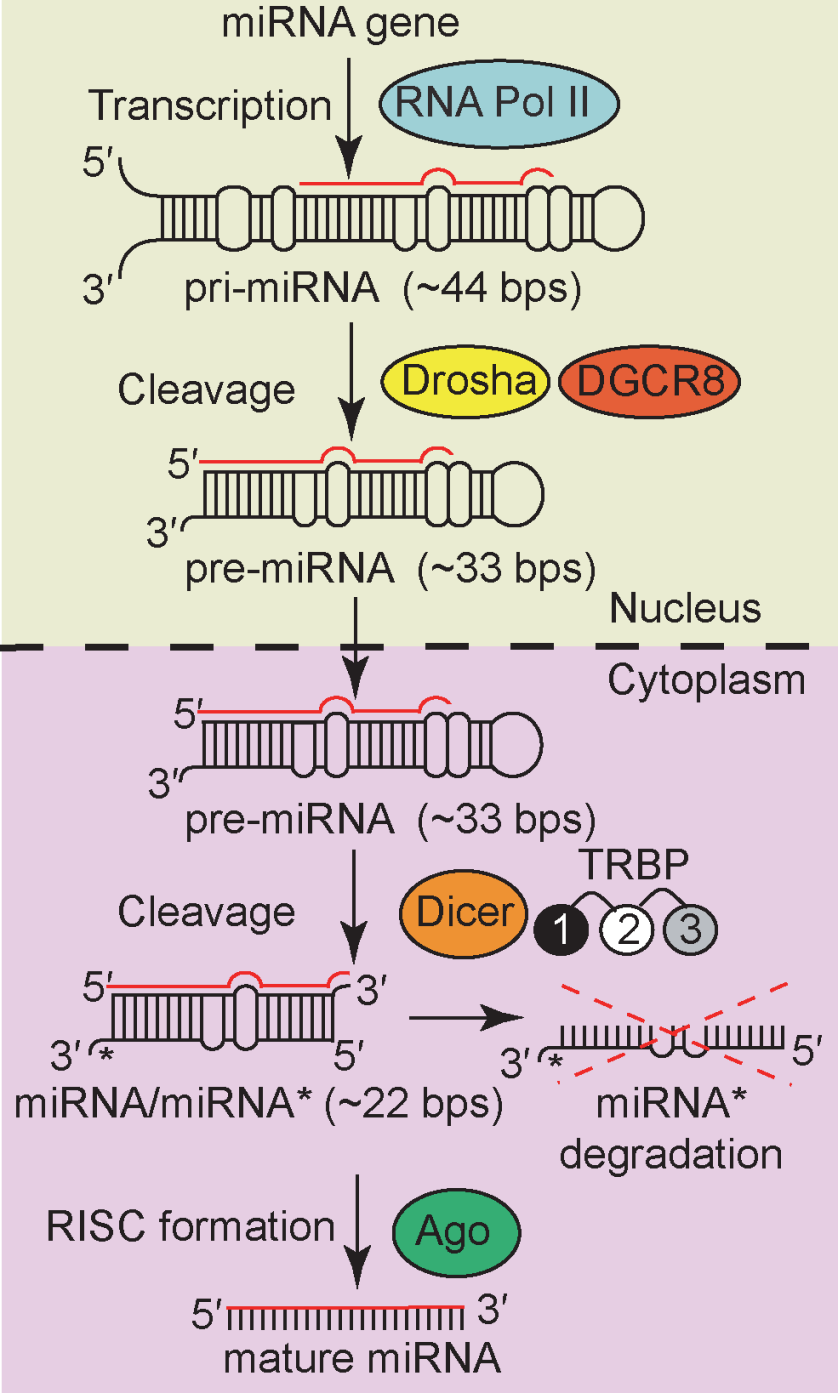
The canonical miRNA maturation pathway. Pri-miRNAs are cleaved by the Microprocessor complex, composed of the enzyme Drosha with DGCR8 as a cofactor, into pre-miRNAs that are subsequently exported out of the nucleus. Further cleavage by the enzyme Dicer with cofactor TRBP produces a miRNA/miRNA* duplex. With the help of Ago, one strand of this duplex is loaded onto RISC to produce the mature miRNA(shown as a solid red line). Note that TRBP is represented as three differently colored dsRBDs, with a grayscale coloring scheme that will be preserved throughout the paper.

Processing of nascent miRNAs to their final form involves two multi-macromolecule complexes (Fig. 1): the ‘Microprocessor’ complex and the RNA-induced silencing complex, RISC, both of which contain an RNase III family enzyme.[14] Specifically, RISC is a dynamically assembled complex requiring a minimum of three proteins: the RNase III enzyme Dicer, one of four Argonaute proteins (AGO1-4), and a double-stranded RNA binding protein (dsRBP) cofactor—TRBP or less frequently PACT.[15] TRBP is a 366-amino acid cytosolic dsRBP implicated in cell growth, spermatogenesis, oncogenesis, and viral replication, due to its role as a cofactor in RISC. In many disease states, cells have been shown to express abnormally high concentrations of TRBP,[16] likely due to its ability to bind directly to PKR, which is responsible for the first steps in innate immunity response.[17]

Nearly all of the proteins named above contain one or more double-stranded RNA binding domains (dsRBDs) and routinely function in gene regulation.[18] One striking feature common to several of the dsRBPs discussed here, including TRBP, is that they contain more than one dsRBD, suggesting that their function and ability to select appropriate binding targets may be enhanced by the co-localization of multiple dsRBDs within a single chain.[19,20] Interestingly, TRBP contains three dsRBDs in tandem, but only the first two (referred to throughout as TRBP-dsRBD1 and TRBP-dsRBD2) have been shown to bind to dsRNA.[21] The third domain, dsRBD3, is mainly involved in protein-protein interactions.[22] Although a general mechanism has not been established, dsRBDs are generally found to discriminate dsRNA based on overall structure and length rather than nucleotide sequence,[23] as has been demonstrated for Dicer, RDE-4, DGCR8, and PKR.[24–27] Several studies have shown that TRBP binds dsRNA in a manner that is independent of dsRNA length;[25,28] however, these studies used much longer RNA molecules that are more commonly encountered in the siRNA maturation pathway, leaving the possibility of length-discrimination in the miRNA maturation pathway unexplored.

This study aims to provide fundamental insights into the molecular mechanism of TRBP binding to RNA commonly encountered in the miRNA maturation pathway, which will lead to a better understanding of its role in delivering mature miRNA to RISC. The findings of this study are motivated through a systematic analysis of TRBP interactions with a model RNA library, monitored by electrophoretic mobility shift assays (EMSAs) and circular dichroism (CD). Our results demonstrate that TRBP is sensitive to imperfections in RNA structure and binds shorter miRNA-relevant length duplexes in a RNA-length dependent manner. One key finding is that the tandem dsRBD-construct of TRBP (TRBP-ΔC) binds dsRNA in a different mode than its isolated dsRBDs. We conclude our study with assays that compare TRBP-ΔC binding to the native pre-mir-16-1 and miR-16-1/miR-16-1* duplex, showing that TRBP is unable to bind to regions of RNA with helical imperfections efficiently and thus is positioned around them. We propose that once positioned by structural defects on the precursor miRNA, TRBP assists Dicer and the rest of RISC in providing efficient and homogenous conversion of substrate pre-miRNA into mature miRNA downstream.

## Materials and Methods

### Protein Preparation

A synthetic TRBP2 (1–345) gene was purchased from Geneart, from which three TRBP constructs were PCR amplified: TRBP-dsRBD1 (9–85), TRBP-dsRBD2 (139–215), and TRBP-ΔC (9–215). The expression and purification of the protein was performed as previously described,[24] with two exceptions. First, protein expression occurred at 24°C over 16 hours. Second, co-purification of endogenous dsRNA required that 5% polyethylenimine (PEI) be added dropwise to the cell lysate until the solution was 0.025% PEI. Following centrifugation, ammonium sulfate was added to the supernatant to a final concentration of 60% ammonium sulfate. The protein was buffer exchanged into 50 mM sodium cacodylate, pH 7.0 and 50 mM potassium glutamate. Sample concentrations were determined via BCA assay (Pierce) or by quantitative FT-IR using an EMD Millipore Direct Detect system (Millipore).

### Mutagenesis

Four triple lysine-to-alanine amino acid mutations were made using a QuickChange Lightening kit (Agilent Technologies) and following the manufacturer’s suggested protocol. The four knockout mutants generated were as follows: TRBP-dsRBD1-Null (K23A, K59A, K60A), TRBP-dsRBD2-Null (K150A, K189A, K190A), TRBP-ΔC-RBD1-Null (K23A, K59A, K60A), and TRBP-ΔC-RBD2-Null (K150A, K189A, K190A). Mutations were verified through DNA sequencing. The expression, purification, and preparation of the mutant proteins were performed as described for the wild-type sequences.

### RNA Preparation

All RNA sequences can be found in S1 Table. DNA for the top and bottom strands encoding the 33 bp and 44 bp duplex RNAs were purchased from IDT. A T7 promoter (5′ to 3′: GAA ATT AAT ACG ACT CAC TAT A) was annealed to the above DNAs to promote T7 transcription in a hemi-duplex method, as previously reported.[29] The RNAs from transcription were purified using a BioLogic LP (BioRad) equipped with an AcroSep Q column (Pall) by equilibrating the column with 15% Buffer B (50 mM Tris pH 7.5, 1 mM EDTA, 2 M NaCl), followed by gradient increase to 65% Buffer B. All non-transcribed RNA strands were purchased from Dharmacon.

In order to ensure proper production of pre-mir-16-1, without T7 artifacts, this RNA was produced by processing pri-mir-16-1 (sequence and *in vitro* transcription conditions reported by Wostenberg et al.[24]) with an *in vivo*-derived ‘Microprocessor’ complex. The Microprocessor (FLAG-Drosha and FLAG-DGCR8 (AddGene)) was expressed and purified as previously described.[30] The lysate was combined with 10 fmol of pri-mir-16-1 RNA, 10X reaction buffer (64 mM MgCl_2_), and RNasin (Promega) and incubated at 37°C for 35 min. The pre-mir-16-1 product was phenol/chloroform extracted from the processing reaction, ethanol precipitated, and purified on a denaturing gel.

### Circular Dichroism

CD measurements of protein-dsRNA binding stoichiometries were performed on a Jasco J-810 spectrometer with a 2 mm path length cuvette at 10°C by titrating increasing amounts of protein into dsRNA following an established protocol.[31] Both the protein and RNA were equilibrated in 50 mM sodium cacodylate pH 7.0, 50 mM potassium glutamate, and 5% glycerol. The increase in ellipticity at 260 nm was measured with equilibration intervals of 7 minutes after addition of each protein aliquot. The starting RNA concentrations ranged from 5–15 μM and the dilution of RNA did not exceed 10% throughout the experiments. The CD titrations were fit to the following expression to obtain the stoichiometry (S):

$$I=I_{o}+(\Delta I/2)\cdot [1+(\frac{\left[P\right]}{\left[R\right]}S)-|1-(\frac{\left[P\right]}{\left[R\right]}S)|]$$ (Eq. 1)

where I is the ellipticity at 260 nm, I_o_ is the ellipticity of naked dsRNA, ΔI is the difference between I and the ellipticity at the saturation point, R is the concentration of dsRNA, and P is the concentration of protein (which varied throughout the experiment). Data fitting was performed in a least-squares manner using MatLab (MathWorks), with the associated error in the fitting represented to two standard deviations. Data points were collected for each length of dsRNA (ds12, ds16, ds22, ds33, ds44) binding each construct (TRBP-dsRBD1, TRBP-dsRBD2, and TRBP-ΔC). Data acquisition for the interaction between the individual dsRBDs and the shorter dsRNA lengths (ds12 and ds16) was attempted, but dsRNA concentrations sufficiently high to ensure that the experiment was performed in the stoichiometric limit saturated the detector. Therefore, the stoichiometry of the individual dsRBDs binding to the ds12 and ds16 duplexes was not established by this assay.

Once the saturating stoichiometries for TRBP-ΔC’s interactions with dsRNA were established as a function of duplex length, the S versus length data were used to determine TRBP-ΔC’s binding site size in base-pairs (*n*) and the maximum extent of overlap between adjacent bound TRBP sites (∂). Binding sites are generally defined by the number of whole integer base-pairs occluded; therefore, we employed a grid search analysis in MatLab that scanned the parameter space, returning integer values for *n* and ∂. Minimization proceeded in a χ^2^ fashion to determine the parameters that best reproduced the experimental S values for the set of RNA lengths, as: [29]

S≤[(m−n)/δ]+1 (Eq. 2)

where the independent variable *m* is the length of the nucleic acid lattices (i.e., the number of base pairs in each RNA duplex.

### Electrophoretic Mobility Shift Assays

For all the EMSAs, the RNA was 5′-end radiolabeled using [γ-^32^P]-ATP. For the duplex RNAs, the radiolabeled top-strand RNA was mixed with a 20-fold molar excess of cold bottom strand and purified as a duplex from an 8% native gel; all self-complementary sequences, including pre-mir-16-1 and the various stem-loop model RNAs, were denatured at 95°C for 1.5 min. and renatured 4°C for 5 min. prior to mixing with protein. EMSAs were carried out as previously described,[24] with the following modifications. Both the protein and RNA were incubated in 50 mM sodium cacodylate, pH 7.0, 50 mM potassium glutamate, and 5% glycerol prior to mixing. The final dsRNA concentration was 0.5 pM in all lanes. Running buffer conditions are 0.25X TBE (25 mM Tris pH 7.0, 20 mM boric acid, 0.25 mM EDTA). Mixing occurred at room temperature for 30 minutes, after which the gel was loaded and run at a constant 200 V for 3.5 h at 10°C.

Gel band intensities of all shifted bands were quantified in ImageQuant and the results were fit to the Hill equation, with the fraction of RNA bound (Θ) values reported representing an average from two independently run gels:

$$\Theta =\Theta_{\max}\cdot [x^{n_{H}}/(K_{d,app}^{n_{H}}+x^{n_{H}})]+\Theta_{\min}$$ (Eq. 3)

where x is the concentration of the protein, K_d,app_ is the apparent macroscopic binding dissociation constant, and n_H_ is the Hill coefficient. In this analysis, K_d, app_, n_H,_ and the lower and upper baselines (Θ_min_ and Θ_max_, respectively) were used as adjustable parameters. Best fit values for these parameters were determined by non-linear least squares analysis, with uncertainties that are reported to two standard deviations.

## Results and Discussion

The dsRBD-containing proteins that permeate the miRNA maturation pathway are highly unlikely to be capable of recognizing RNA molecules by nucleotide sequence, suggesting that they must select target RNA molecules through recognition of other structural features. A previous study by Parker et al.[25] probed the mechanism of TRBP binding long dsRNA (40–650 bps) and found that TRBP binds to these dsRNAs with similar macroscopic K_d,app_ values, indiscriminate of length. The study showed that TRBP gave banding patterns featuring multiple intermediates with varying mobilities in gel shift assays, particularly at non-saturating TRBP concentrations. Here, we have designed a study featuring shorter dsRNAs, aiming to better understand how TRBP interacts with miRNA-relevant RNAs, as TRBP may engage in fundamentally different binding modes for these two regimes.

Prior data shows that the dsRBD from the RNase III enzyme Dicer (the enzyme for which TRBP acts as a cofactor) binds to both the naturally occurring pre-mir-16-1 and a Watson-Crick (W-C) duplex of the same length with similar macroscopic binding affinities,[29] suggesting that the use of W-C duplexes as models for dsRBD-dsRNA interactions can lead to useful insights for biologically relevant sequences. Therefore, we began our study with various lengths of W-C duplex RNAs specifically designed to test if TRBP is capable of distinguishing between pre-miRNA (Dicer substrate) and the shorter miRNA/miRNA* duplex (Dicer product). In particular, the ds44 and ds33 duplex lengths approximately correspond to typical pri-miRNA and pre-miRNA lengths, ds22 to miRNA/miRNA* duplex length, ds16 to the smallest length for dsRNA seen biochemically, and ds12 to the smallest length seen to bind dsRBDs crystallographically. Using these RNAs, we sought to characterize the macroscopic stoichiometries and affinities responsible for TRBP-dsRNA interactions, through a combination of CD spectroscopy and EMSAs. Three constructs of TRBP were produced (TRBP-dsRBD1, TRBP-dsRBD2, and TRBP-ΔC) so that mechanistic effects induced by interactions between multiple dsRBDs within a single polypeptide chain could also be defined.

### The stoichiometry of TRBP-ΔC binding to dsRNA increases step-linearly with duplex length

**Figure 2:**
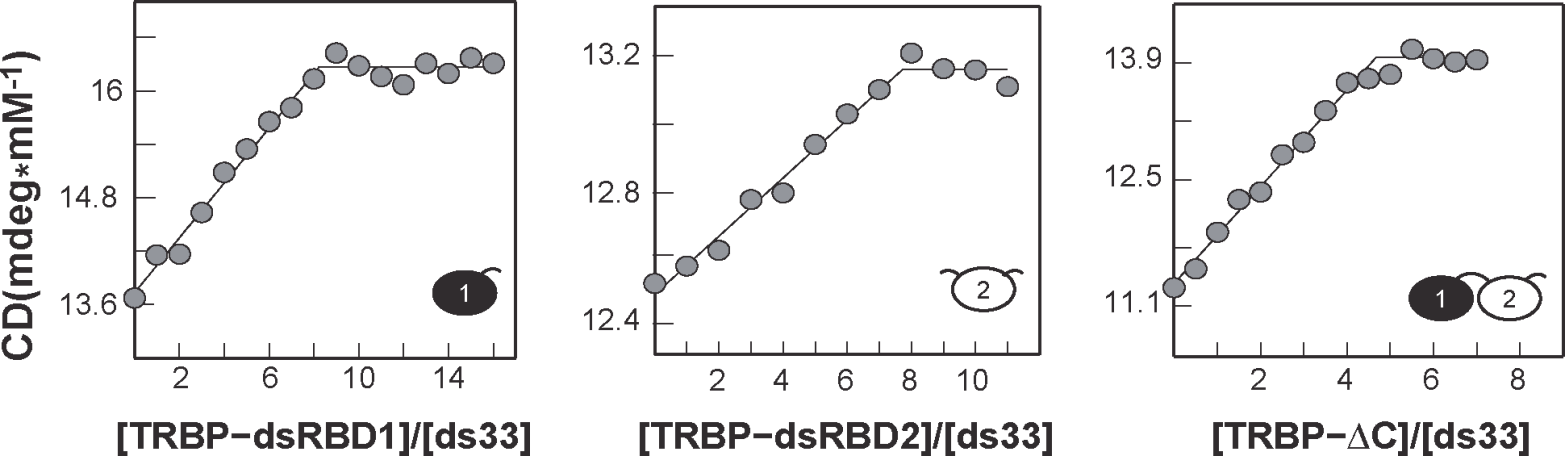
Determination of TRBP binding stoichiometry for ds33 by circular dichroism. Stoichiometric amounts of TRBP constructs were manually titrated into a solution of ds33 at 10°C. Each data point (*grey dots*) represents an average of three consecutively taken measurements. The best-fit line from Equation 1 (*black line*) shows a binding stoichiometry of approximately 7–8 dsRBDs for TRBP-dsRBD1 and TRBP-dsRBD2, and approximately 5 TRBP-ΔC molecules per molecule of ds33.

It has been shown that an increase in dsRNA’s circular dichroic signal at 260 nm correlates with dsRBD interaction.[32] As such, CD has been used to characterize the stoichiometry of PKR binding to dsRNA, yielding quantitatively similar results to those obtained by analytical ultra-centrifugation.[31] Therefore, we performed CD titrations as a means of determining the saturating stoichiometry of TRBP binding to our set of RNA duplexes. To approach the stoichiometric limit without saturating the CD detector, TRBP was titrated into 5–15 μM dsRNA to final concentrations of at least 7-fold molar excess over the K_D_ determined by EMSA (*vide infra*). Fig. 2 shows the result of titrating excess TRBP into ds33 (full data series presented in S1 Fig.). The best-fit line to Equation 1 (Experimental Procedures) reveals that the isolated dsRBD constructs (TRBP-dsRBD1 and TRBP-dsRBD2) bind with a stoichiometry of approximately eight dsRBDs to a single ds33 molecule, while TRBP-ΔC binds with a stoichiometry of approximately five TRBP-ΔC molecules to a single ds33 molecule (see tabulated results for all TRBP constructs in Table 1). Encouragingly, the results for TRBP-ΔC saturating ds22 corroborate the apparent stoichiometry of 2:1 seen for TRBP-ΔC binding a 21-mer dsRNA derived from ITC data presented by Yamashita et al.[33] Overall, the stoichiometry results suggest that TRBP employs an alternative binding mode in which it is able to place more dsRBDs onto an RNA of a given length than its isolated dsRBD constructs, which could explain the selective pressure to maintain multiple copies of dsRBDs in a single polypeptide chain.

**Table 1:** Stoichiometries for TRBP constructs binding various dsRNA lengths.

**RNA construct**	**ds12**	**ds16**	**ds22**	**ds33**	**ds44**
**[RNA] μM**	15.0	13.8	8.0	6.8	5.0
**TRBP-dsRBD1**	N/D	N/D	5.0±1.0	7.4±0.7	7.5±0.5
**TRBP-dsRBD2**	N/D	N/D	5.0±1.0	7.7±0.6	7.1±1.0
**TRBP-ΔC**	0.8±0.1	0.8±0.1	2.2±0.3	4.7±0.2	5.5±0.3

**Figure 3:**
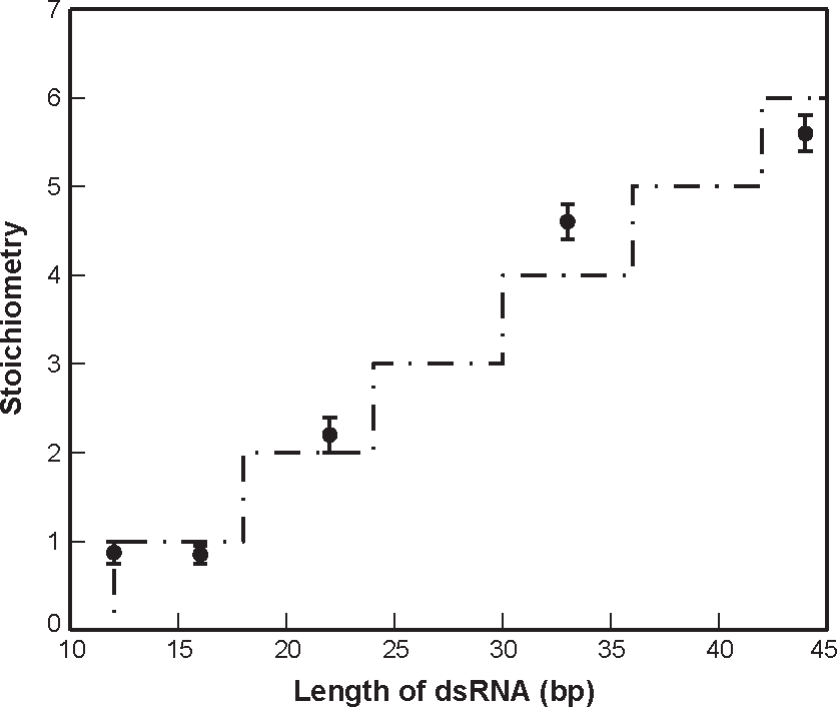
The characteristics of TRBP-ΔC binding sites on W-C dsRNA lattices are revealed by analyzing variation in the stoichiometries established by CD. Each data point (*black dots*) represents the stoichiometry for TRBP-ΔC binding a particular dsRNA. A grid search was conducted to simultaneously yield the binding footprint (*n*, in base pairs) and allowable site overlap (δ, in base pairs). The best-fit line to Equation 2 (*dot-dash*) was produced with *n* = 12 bps and δ = 6 bps.

Crystallographic data of individual dsRBDs bound to dsRNA suggest that dsRBDs typically occupy a binding footprint of 12–16 bps along one face of the dsRNA.[34] If we assume that binding only occurs on one face of the dsRNA, inspection of the stoichiometry results from CD forces one to the conclusion that the TRBP-ΔC binding footprint must decrease as the dsRNA length decreases (from 12 bps to 8 bps), which is unlikely. In reality, it is possible for the dsRBDs to occupy partially overlapping binding sites around the RNA, as the A-form helix is a 3-dimensional object. Therefore, we performed global analysis of the CD data for TRBP-ΔC that allowed us to simultaneously determine the binding site size (n) and the amount of overlap required (δ) to accommodate the observed saturating stoichiometry of TRBP-ΔC molecules for each given RNA length. Fitting the stoichiometry data as a function of dsRNA length (Fig. 3) demonstrates that, for all dsRNA lengths investigated, TRBP-ΔC binds dsRNA with a binding footprint of 12 bps and an overlap size of 6 bps. The results also suggest that the number of TRBP-ΔC molecules that bind dsRNA grows step-linearly at an increment of approximately one TRBP-ΔC molecule per turn of added A-form helix to a dsRNA length of at least 44 bps, the longest length used in this study.

### The binding affinity of TRBP increases with increasing RNA duplex length

**Figure 4:**
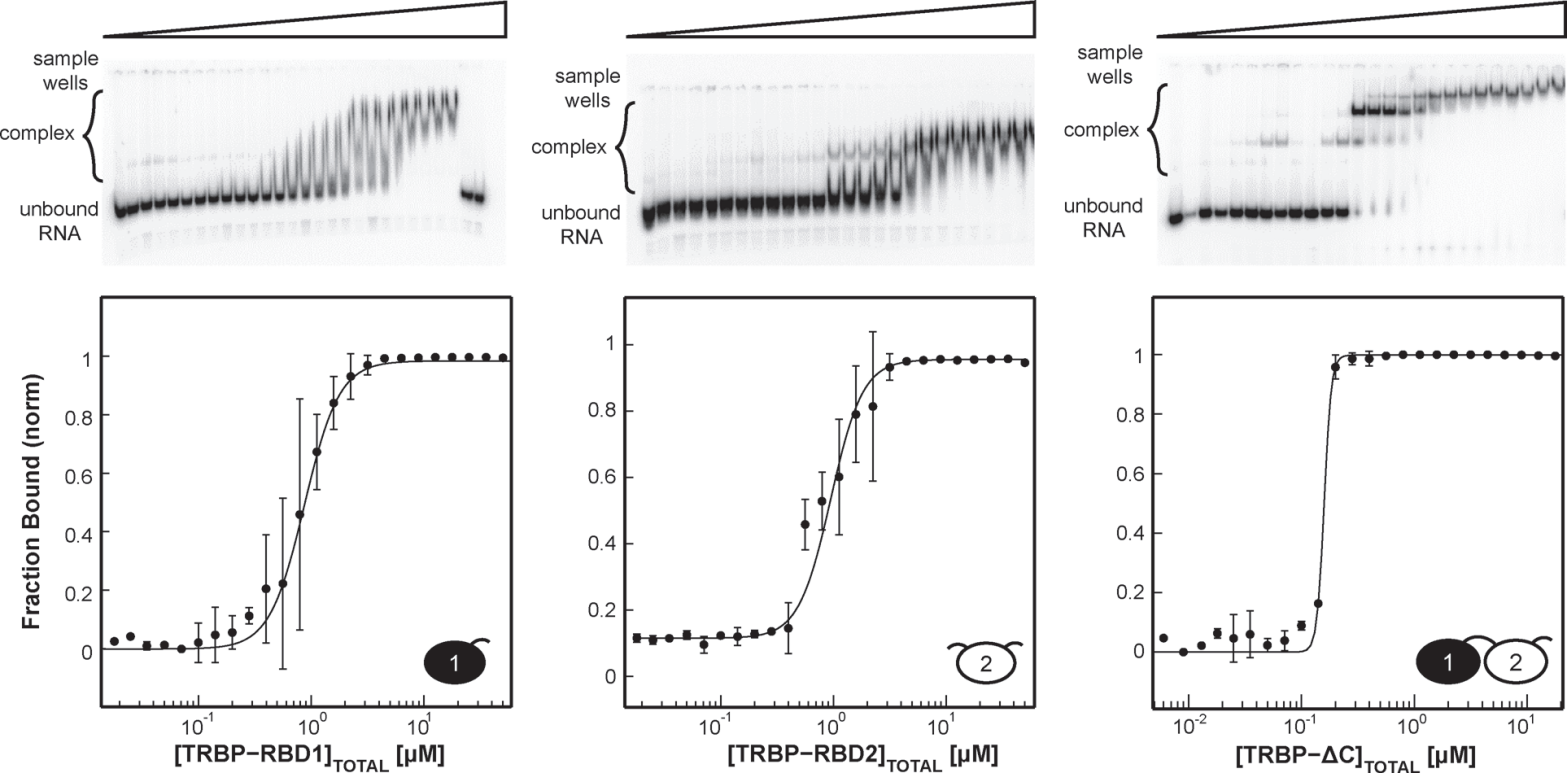
EMSA results for constructs of TRBP binding ds33. The radiograph image of a representative gel is presented, with the bar above it representing the increase in [TRBP] from left to right. Below the gel is the Hill analysis of a set of two titrations. The experimental data (*black dots*) are averaged from the two independent experiments, with the black best-fit line produced from the best-fit parameters reported in Table 3. It is interesting that TRBP-ΔC binds dsRNA with ∼3-fold tighter macroscopic binding affinity than its individual dsRBDs.

**Figure 5:**
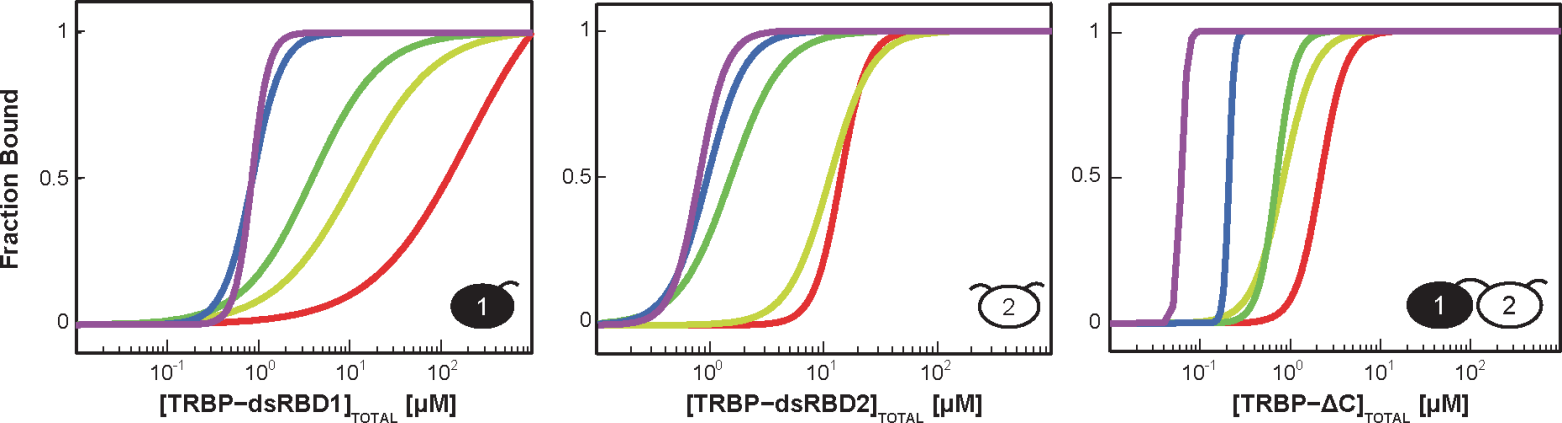
Macroscopic analysis of EMSA data for TRBP’s dsRBDs reveals length-dependent affinity for dsRNA. The fits for each dsRNA length are shown in a rainbow color array with ds12 in purple, ds16 in blue, ds22 in dark green, ds33 in light green and ds44 in red. For all TRBP constructs, K_d,app_ changes approximately 4- to 6-fold between ds22 and ds33/ds44, which corresponds to the lengths of its substrate and product. Also noteworthy is that for all RNA duplex lengths, TRBP-ΔC binds dsRNA with ∼10-fold tighter macroscopic binding affinity than its individual dsRBDs.

Having established that the saturating TRBP stoichiometry on dsRNA grows with duplex length, we next tested the hypothesis that TRBP binding affinity increases with dsRNA length when the RNAs considered are of a size relevant to processing in the miRNA maturation pathway. Representative gels shown in Fig. 4 highlight the results of binding TRBP’s individual dsRBDs and TRBP-ΔC to ds33 (all results tabulated in Table 2). Best-fit macroscopic apparent dissociation constants (K_d,app_) of TRBP-dsRBD1 (0.9±0.3 μM), TRBP-dsRBD2 (1.00±0.08 μM), and TRBP-ΔC (0.20±0.02 μM), suggest that by having tandem dsRBDs, TRBP-ΔC increases its apparent binding affinity for ds33 by approximately 5-fold over the individual dsRBD constructs. Table 2 demonstrates that for both the individual dsRBDs and the TRBP-ΔC construct, K_d,app_ increases as the length of dsRNA increases, but that the changes are relatively small (representative gels for binding to each RNA length are displayed for TRBP-dsRBD1, TRBP-dsRBD2, and TRBP-ΔC in S2–S4 Figs., respectively). Of particular interest is the gap seen for TRBP-ΔC binding to dsRNA matching the length of its substrate (pre-miRNA, ∼33 bps) and its product (miRNA/miRNA* duplex, ∼22 bps). Over this range, the K_d,app_ changes by approximately 3-fold (Fig. 5), implying that TRBP possesses minimal ability to differentiate between pre-miRNA and miRNA/miRNA* duplexes based on length alone.

**Table 2:** Best fit Macroscopic binding affinities and Hill Coefficients from EMSA data.

**W-C duplex RNAs: K_d,app_(μM)**					
**RNA construct**	**ds12**	**ds16**	**ds22**	**ds33**	**ds44**
**TRBP-dsRBD1**	N/D	>15	3.5±0.2	0.9±0.3	0.8±0.05
**n_H_**	N/D	>1	1.5±0.2	2.9±0.7	2.6±0.3
**TRBP-dsRBD2**	N/D	>11	1.7±0.1	1.00±0.08	0.80±0.06
**n_H_**	N/D	>1	2.7±0.5	3.1±0.5	4.3±0.8
**TRBP-ΔC**	2.0±0.3	0.80±0.07	0.60±0.05	0.20±0.02	0.06±0.01
**n_H_**	1.9±0.2	3.2±0.9	4±0.7	>12	>14
**Ds16 with varying loop lengths: K_d,app_(μM)**					
**RNA construct**	**ds16 (no loop)**	**stable tetra**	**tetra-U**	**hexa-U**	**octa-U**
**TRBP-dsRBD1**	>15	9±1	7.0±0.9	10±3	8±3
**n_H_**	>1	1.3±0.3	1.0±0.1	1.0±0.1	1.3±0.3
**TRBP-dsRBD2**	>11	5.0±0.7	4.0±0.9	>4	>6
**n_H_**	>1	2.4±0.3	2.7±0.8	>2	>2
**TRBP-ΔC**	0.80±0.07	0.50±0.05	0.70±0.05	0.61±0.05	0.41±0.05
**n_H_**	1.4±0.2	1.1±0.1	4.1±0.5	3.1±0.6	2.5±0.2
**Native sequences: K_d,app_(μM)**				
**RNA construct**	**pre-mir-16-1**	miR-16-1/miR-16-1*	**A-mismatch**	**U-bulge**
**TRBP-ΔC**	0.61±0.02	1.10±0.01	1.78±0.08	0.30±0.03
**n_H_**	1.6±0.1	1.1±0.2	2.7±0.2	5±1

Next we attempted to determine whether the terminal loop present in pre-miRNA affects TRBP binding to dsRNA. To carry out this study, we chose ds16 as the base duplex because CD demonstrated a 1:1 stoichiometry for this stem length, which renders our results free from convolution artifacts arising from some copies of TRBP binding proximal to the terminal loop, while others engage the distal portion of the RNA with a potentially distinct binding constant. Four stem-loops were chosen: a thermally stable tetra-loop (UUCG), tetra-U, hexa-U, and octa-U loops (representative gels for binding TRBP-dsRBD1, TRBP-dsRBD2, and TRBP-ΔC to each these loop constructs are displayed in S5–S7 Figs., respectively). Table 2 shows that all TRBP constructs bound these four loop constructs with essentially equal affinity (within experimental uncertainty), which suggests that TRBP is insensitive to loop structure. Taken together, our data suggest that while TRBP may be able to discriminate weakly between pre-miRNA and miRNA/miRNA* duplexes based on length, it is unlikely that TRBP senses the presence of the terminal loop in pre-miRNA.

### Importance of multi-dsRBDs: Sensing imperfections in miRNA

**Figure 6:**
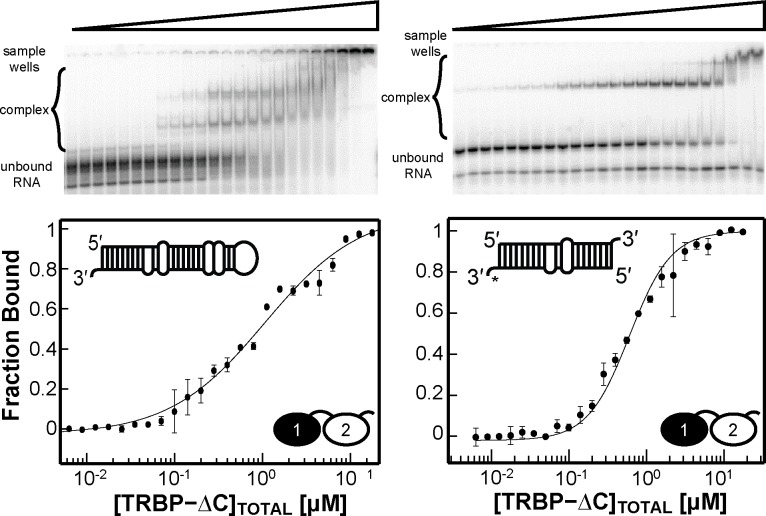
EMSAs results for TRBP-ΔC binding to substrate pre-mir-16-1 (*left*, and natural product miR16-1/miR16-1* duplex (*right*). The experimental data (*black dots*) are averaged from two independent experiments, with the black best-fit line produced from the best-fit parameters reported in Table 3. The minimal difference in binding affinity between substrate and product (< 2-fold) suggests that RNA length is not the sole parameter to consider when determining TRBP binding affinity.

TRBP serves multiple functions in the context of RNAi. For example, *in vivo* assays show that TRBP is required for Dicer to more efficiently and homogeneously cleave pre-miRNA.[35,36] TRBP may also be responsible for guide strand selection in RISC, where it recognizes terminal mismatches at the ends of siRNA.[15] Furthermore it has been suggested that these imperfections lead to tighter binding by TRBP and subsequently higher efficiency downstream; contrastingly, the presence of terminal mismatches can also decrease TRBP binding affinity leading to lower processing efficiency.[37] Therefore, having established the influence of duplex length and terminal loop structure on TRBP binding to dsRNA, we next attempted to characterize the impact of helix imperfections by monitoring binding of TRBP to a native miRNA duplex. Differences with respect to our idealized W-C RNA constructs may arise with native duplexes, because pre-miRNA stems are highly enriched in bulges and non-canonical base pairs that may perturb TRBP-dsRNA interactions.[38] We have previously studied Dicer-dsRBD binding to pre-mir-16-1 and so chose this sequence, as well as the miR-16-1/miR-16-1* duplex, for the basis of this study. The EMSA results for the TRBP-ΔC construct binding pre-mir-16-1 and the miR-16-1/miR-16-1* duplex are displayed in Fig. 6 with best-fit values using the Hill analysis provided in Table 2. Based on the results of our length-dependence study, we expected pre-mir-16-1 to bind with a K_d,app_ similar to binding ds33 and the miR-16-1/miR-16-1* complex with a K_d,app_ similar to binding ds22. Rather, the results show that TRBP-ΔC binds pre-mir-16-1 and the miRNA/miRNA* duplex with weaker affinities (K_d,app_ = 0.61±0.02 μM and 1.1±0.1 μM, respectively), that do not match predictions based on their respective total lengths in base pairs. While the above results is inconsistent with our initial prediction, Benoit et al. reported similar binding affinities for pre-mir-155 and the miR-155/miR-155* duplex using ITC,[39] suggesting that our observation may be general.

Both the substrate and product miRNA contain helical imperfections; Mfold analysis of pre-mir-16-1 predicts a 1nt U-bulge, two bases removed from a thermodynamically unstable A•A mismatch, located central to the stem of the RNA, which Quarles et al.[30] suggest deforms the A-form helical structure. Therefore, both of the binding affinities seen for pre-miRNA and miRNA/miRNA* duplexes are reasonable if TRBP-ΔC is sensitive to helical imperfections (e.g., internal loops and bulges). The deformations break both pre-mir-16-1 and the miRNA/miRNA* duplex into two pseudo-stem regions, suggesting the hypothesis that modelling pre-mir-16-1 as two 20-mer stems and miR-16-1/miR-16-1* as two 10-mer stems will bring our results into accord with the expectation values established from our simple length-dependence study. Inspection of the dissociation constants in Table 2 confirms this to be a reasonable model. Therefore, we propose the general hypothesis that imperfections along the A-form RNA, which are nearly always found in both pre-miRNA and miRNA/miRNA* duplexes,[40] influence the ability of TRBP to bind efficiently.

### Helical defects in dsRNA influence multi-dsRBDs binding affinity

**Figure 7:**
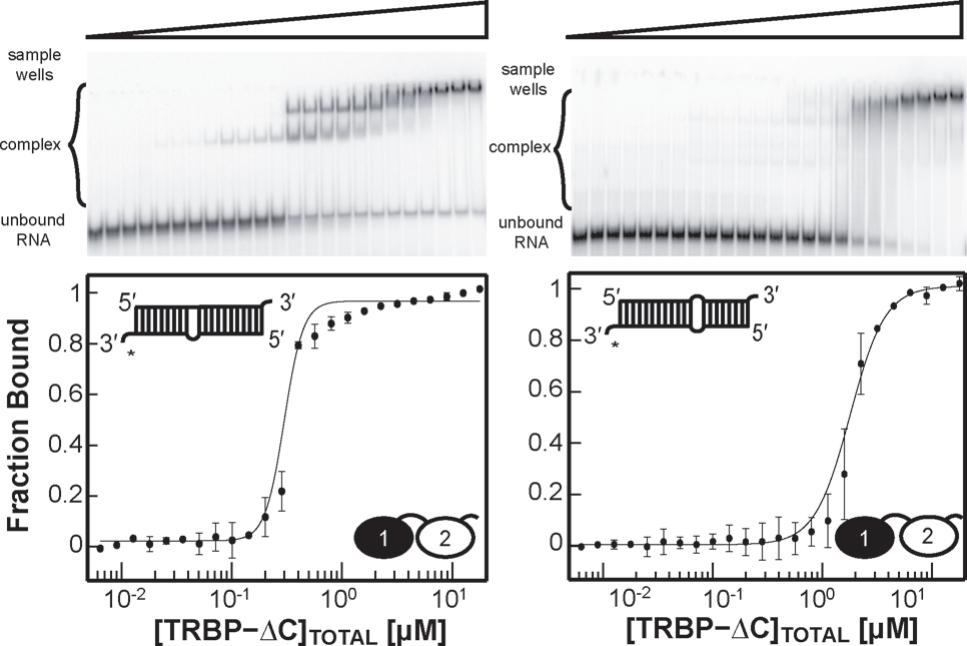
EMSAs results for TRBP-ΔC binding constructs that mimic miR16-1/miR16-1* imperfections: a 1nt U-bulge RNA (*left*), and A•A mismatch (*right*). The experimental data (*black dots*) are averaged from two independent experiments, with the black best-fit line produced from the best-fit parameters reported in Table 2. Both constructs have the same overall length, suggesting that the 2-fold change in binding affinity between ds22 and both pre-miR16-1 and the miR/miR* duplex is due to the 1nt U-bulge and not the A•A mismatch.

TRBP expressed the same binding affinity for both pre-miRNA and miRNA/miRNA* duplexes, despite the difference in overall RNA length. Given the results that TRBP binding is not affected by the addition of terminal loop, we chose to test TRBP binding to RNA duplex constructs that bore either the 1nt U-bulge or the A•A mismatch in the context of a 22 bp W-C duplex based off of the miR16-1/miR16-1* duplex (sequences shown in S1 Table). The results of the RNA deformation study are presented in Fig. 7, along with representative structures, with the corresponding binding affinities presented in Table 2. While the difference in binding affinity between a TRBP-ΔC binding ds22 and the miR/miR* construct is minimal, it is very interesting that this difference is maintained between the A•A mismatch and the 1 nt U-bulge construct (K_d,app_ = 1.78±0.08 μM and 0.30±0.03 μM, respectively). The results suggest that possessing a 1×1 nt A•A mismatch in miRNA is sufficient to change the observed binding affinity of TRBP-ΔC. Surprisingly a 1×0 nt U-bulge construct, which may be expected to kink the lowest free energy conformation of the duplex, had no deleterious effect in the observed binding affinity. Therefore, we conclude that imperfections in the W-C region of the dsRNA stem establish the relevant length scale for substrate selection. Given the prevalence of imperfections in microRNA denoted in miRBase,[40] we hypothesize that the total length of the pre-miRNA or miRNA/miRNA* stem will rarely establish the affinity for TRBP binding. We anticipate that the trends in binding affinities we observe for TRBP-ΔC interactions with pre-mir-16-1 and the miR-16-1/miR-16-1* duplex are general and will impact TRBP’s interactions with the entire cellular pool of miRNA precursors.

### Domain Knockouts reveal functional connections between dsRBD1 and dsRBD2 in TRBP-ΔC

Motivated by the observation that TRBP-ΔC binds to RNA differently from the mode of binding employed by either of its two dsRBDs in isolation, we next aimed to test the hypothesis that RNA engagement by both dsRBDs in TRBP-ΔC is necessary to create the observed binding mode and preference for uniform Watson-Crick dsRNA segments. At present, there are no co-crystal structures of tandem dsRBD proteins bound to dsRNA to use for rational design of this experiment; however, it is well known from the set of available dsRBD-dsRNA co-crystal structures that the poly-lysine motif on helix-2 of the dsRBD is a critical component of the dsRNA binding surface. Therefore, following the model of Sohn et al., established for the Drosha cofactor protein DGCR8, we knocked out dsRBD binding activity in each of the two dsRBDs of TRBP through the generation of triple lysine-to-alanine substitutions in helix-2. RNA-binding activity knockouts of dsRBD1 (TRBP-dsRBD1-Null) and of dsRBD2 (TRBP-dsRBD2-Null) were confirmed by EMSA, where no binding to ds22 was observed (S8 Fig.). The null-activity mutants of each dsRBD were then made in the context of TRBP-ΔC, yielding the constructs TRBP-ΔC-RBD1-Null and TRBP-ΔC-RBD2-Null for further study.

**Figure 8:**
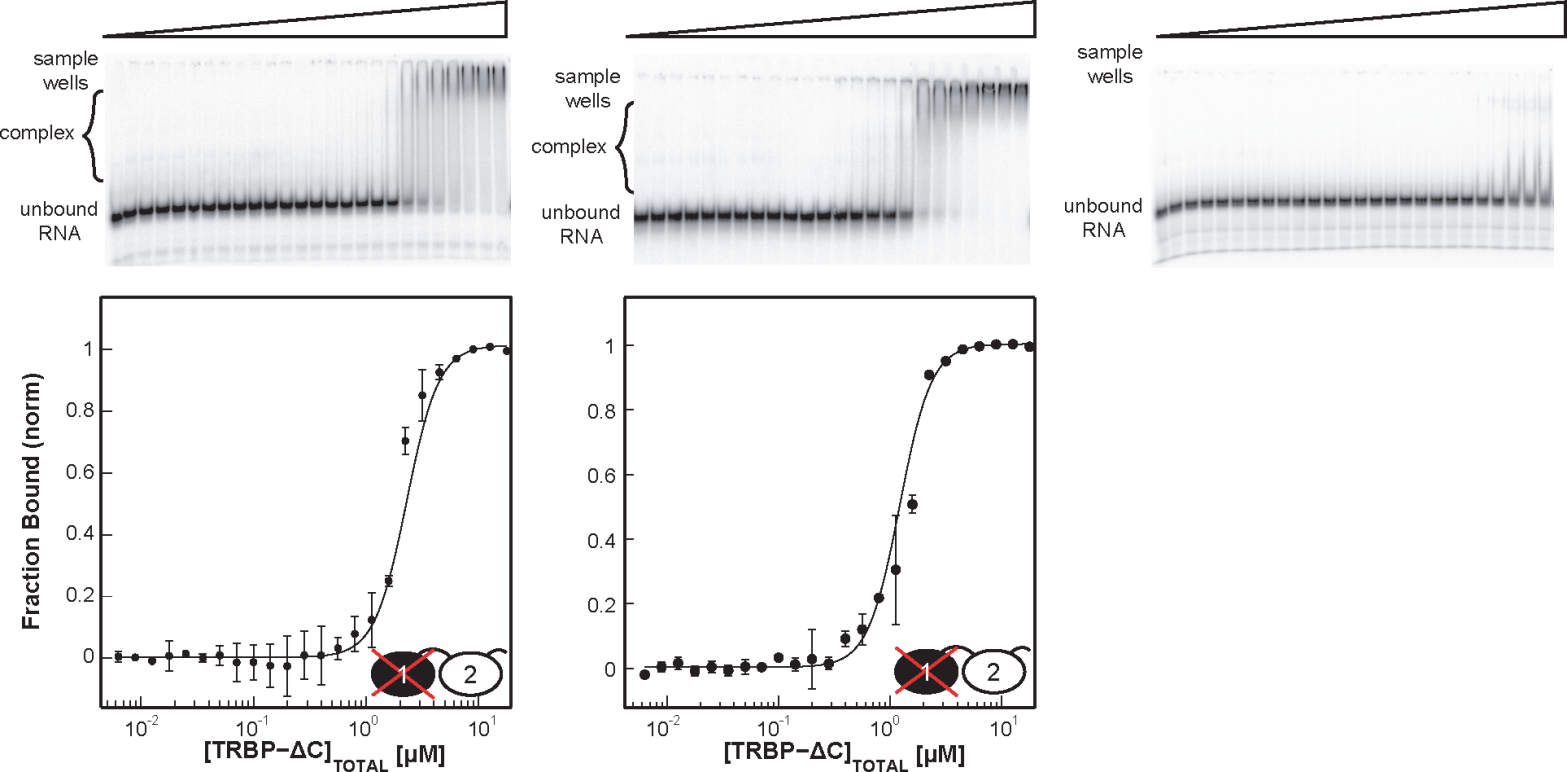
EMSAs results for TRBP-ΔC-RBD1-Null binding ds22, ds33, and miR16-1/miR16-1* from left to right. The experimental data (*black dots*) are averaged from two independent experiments, with the black best-fit line produced from the best-fit parameters reported in Table 3. Binding affinities for ds22 and ds33 correlate well with those of the individual TRBP-RBD2 construct.

**Figure 9:**
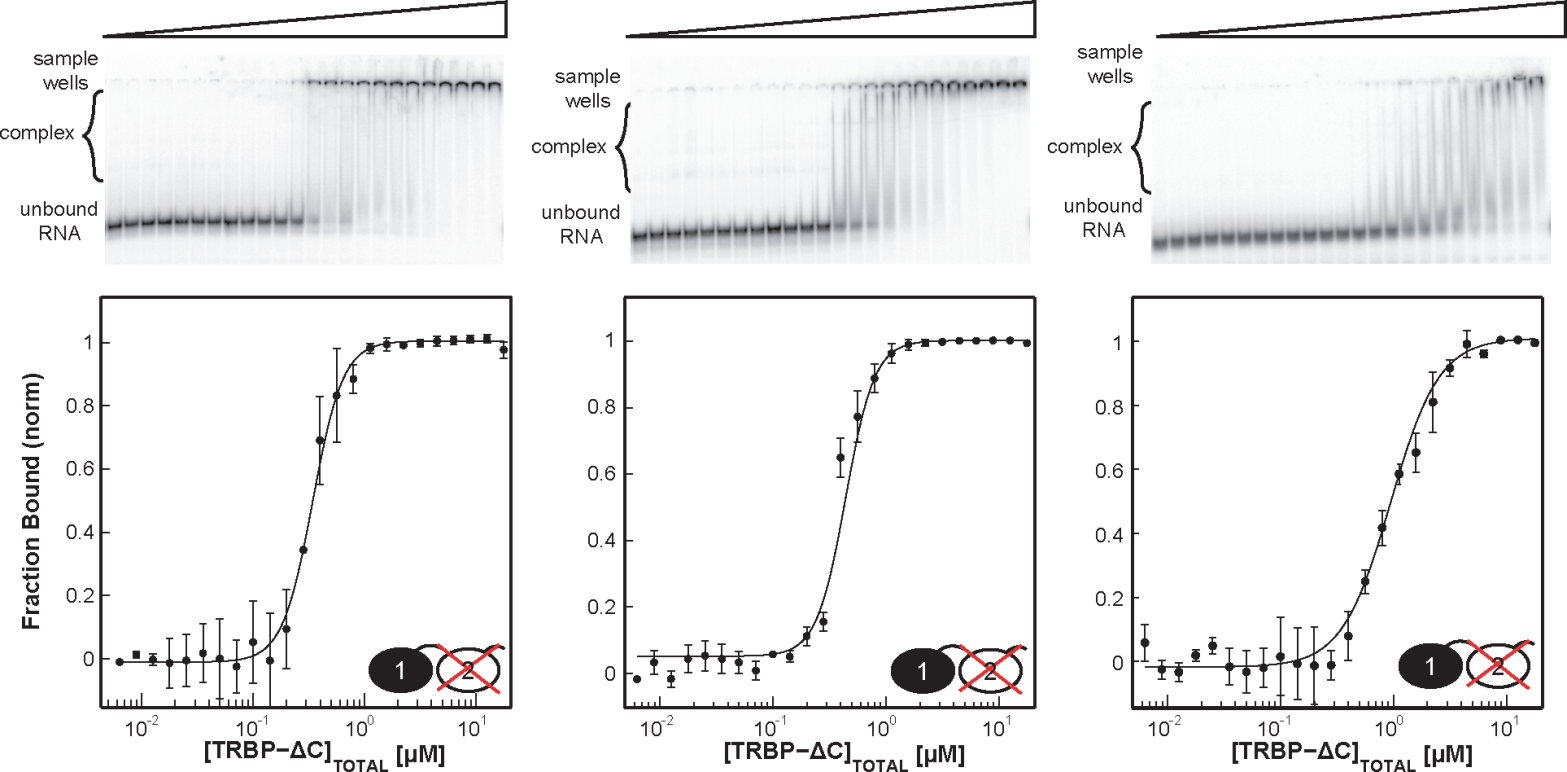
EMSAs results for TRBP-ΔC-RBD2-Null binding ds22, ds33, and miR16-1/miR16-1* from left to right. The experimental data (*black dots*) are averaged from two independent experiments, with the black best-fit line produced from the best-fit parameters reported in Table 3. Interestingly the binding affinities for ds22, ds33, and miR16-1/miR16-1* correlate well with those of individual TRBP-ΔC and not of the individual TRBP-RBD1 construct.

The two TRBP-ΔC domain knockout mutants were used in EMSA assays to test for binding to ds22, ds33, and the miR-16-1/miR-16-1* duplex. Representative gels from the study of TRBP-ΔC-RBD1-Null and TRBP-ΔC-RBD2-Null are displayed in Fig. 8 and Fig 9, respectively, with a summary of the best-fit apparent dissociation constants and hill coefficients in Table 3. Knocking out dsRBD1 binding activity in the TRBP-ΔC context produced a protein that binds to each of these dsRNAs with a similar affinity to that recorded for TRBP-dsRBD2 alone (compare Tables 2 and 3), although with a different band pattern observed in the gels (compare Fig. 4 with Fig. 8). Surprisingly, knocking out dsRBD2 activity in the context of TRBP-ΔC had almost no impact on the apparent dissociation constant for these three dsRNAs (compare Tables 2 and 3), although the pattern of bands observed on the gel was again perturbed, relative to wild type (compare Fig. 4 with Fig. 9).

**Table 3:** Best fit macroscopic binding affinities and Hill Coefficients from EMSA analysis of TRBP mutants.

**Mutant Studies: K_d, app_(μM)**			
**RNA construct**	**ds22**	**ds33**	miR-16-1/miR-16-1*
**TRBP-dsRBD1-Null**	N/D	--	--
**TRBP-dsRBD2-Null**	N/D	--	--
**TRBP-ΔC1-Null**	2.29±0.08	1.22±0.07	N/D
**n_H_**	3.1±0.2	3.0±0.4	--
**TRBP-ΔC2-Null**	0.34±0.01	0.44±0.05	0.9±0.1
**n_H_**	3.2±0.6	3.4±0.7	2.0±0.4

Evaluation of the prior literature for the tandem-dsRBD proteins DGCR8 and PKR offers some insight into these results. Sohn et al. reported triple lysine/arginine-to-alanine substitutions for DGCR8 producing constructs they name DGCR8-D1H2 (equivalent to TRBP-ΔC-RBD1-Null) and DGCR8-D2H2 (equivalent to TRBP-ΔC-RBD2-Null), for which they report binding assays in Fig. 2 of their paper.[20] Although the Sohn et al. study did not include measurements performed on the individual dsRBDs from DGCR8, these data have been reported by Wostenberg et al. for DGCR8-dsRBD1, enabling comparison. No such comparative data can be evaluated for DGCR8-dsRBD2, which does not appear to be soluble in the absence of dsRBD1 (KAQ and SAS, unpublished). When DGCR8-dsRBD2 activity is knocked out, the apparent dissociation constant for DGCR8-Core binding to pri-mir-16-1 changes from 2.1 ± 1.1 μM to 8.2 ± 1.2 μM.[20] Comparing to this, Wostenberg et al. report pri-mir-16-1 apparent dissociation constants of 3.7 ± 0.1 μM and 9.4 ± 0.4 μM for DGCR8-Core and DGCR8-dsRBD1, respectively.[24] Thus, the results we present here for knockout of dsRBD1 in the context of TRBP-ΔC are similar to those previously reported for domain knockout in DGCR8-Core. Conversely, when Sohn et al. knock out dsRBD1 activity they observe the same magnitude of reduction in dsRNA binding affinity as they observed for knockout of dsRBD2,[20] which contrasts with our observation of in-equivalent effects from knocking out dsRBD1 and dsRBD2 in TRBP.

DGCR8-Core is dissimilar from TRBP-ΔC and PKR-dsRBD in two significant ways. Structurally, the two dsRBDs of DGCR8 are observed to form a stable and RNA-independent interaction with each other, whereas both TRBP-ΔC and PKR have been shown by NMR spectroscopy to have two non-interacting dsRBDs (in the absence of RNA), separated by a flexible polypeptide linker.[28,41] Second, the affinity of DGCR8-Core for dsRNA is only 2- or 3-fold stronger than the comparable affinities of the isolated DGCR8-dsRBD1.[24] In contrast to this, TRBP-ΔC displays an approximately 10-fold affinity enhancement over the individual dsRBDs (this study) and PKR an approximately 30-fold enhancement.[31] Significantly, NMR studies of PKR binding to dsRNA showed that the N-terminal dsRBD of PKR engages dsRNA extensively, with only minimal additional contacts contributed by the C-terminal dsRBD in the context of short dsRNA,[31] comparable to those we have investigated in this study. In contrast, NMR studies of DGCR8-Core interaction with dsRNA showed extensive chemical shift perturbations spanning both dsRBDs.[41] Consideration of our TRBP-ΔC-RBD2-Null results in this context leads us to the hypothesis that the dsRNA binding mode employed by TRBP is highly similar to the binding mode employed by PKR for engagement of short dsRNAs on the length-scale of pre-miRNA and miR/miR* duplexes. This intriguing possibility will be the subject of future study.

## Conclusions

Generally, dsRBDs do not display sequence-dependent RNA binding, but are often shown to bind in a length-dependent manner; in contrast, TRBP had previously been shown to bind in a length-independent manner using siRNA mimics.(22) Although this may be the case for siRNA maturation, the results presented here suggest that this is not so for human TRBP engaging shorter miRNA-related RNA. For short dsRNA, we observe that as the length of the dsRNA increases up to 44 bp, TRBP’s binding affinity increases (Table 2). Intriguingly, we observe that for the longer length dsRNAs in our study, the change in apparent dsRNA binding affinity begins to taper off (Fig. 5), which if extrapolated to longer dsRNA could lead to similar macroscopic binding affinities regardless of dsRNA length. By combining our results and those published by Parker et al., [25] we see that TRBP may use duplex-length as a means to differentiate between various potential dsRNA targets. Thus, our data suggest that TRBP engages dsRNA in a mechanistically distinct manner for short dsRNA (i.e., miRNA precursors), as compared to long dsRNA (i.e., siRNA precursors). Intriguingly, our dsRBD knockout studies suggest that a more direct comparison for the dsRNA binding mechanism employed by TRBP to engage miRNA-precursors may be the short dsRNA mode of PKR, in which interactions are dominated by the N-terminal dsRBD, with enhancement by the C-terminal dsRBD.

It is intriguing that the process of maturing cellular pools of hundreds of miRNAs with distinct sequence compositions is carried out through interactions with enzymes and cofactors that rely heavily on sequence-independent dsRBDs for substrate selection. The results of this study show that the multi-dsRBD containing protein, TRBP, is capable of discriminating dsRNA with lengths spanning the range corresponding to its substrate and product RNAs in the miRNA maturation pathway. Investigating how TRBP binds to native sequences, such as pre-mir-16-1 and the miR-16-1/miR-16-1* duplex, was facilitated by our length-dependent study of TRBP binding W-C duplex RNAs, combined with the stem-loop dependent study. More importantly, we demonstrate here that TRBP’s binding affinity to dsRNA is affected by the presence of helical imperfections ubiquitous in miRNA precursors, suggesting that the RNA itself may hold the key to maintaining cut site fidelity in a pathway dominated by non-sequence specific binders. The combined results suggest that *in vivo*, TRBP’s role in miRNA maturation may be to sense and bind around the abundant imperfections in substrate miRNA precursors, thereby helping to establish the cut site position for cleavage by Dicer.

## Supporting Information

S1 Table Sequences of RNA Oligonucleotides used in this study. (DOCX)

S1 Fig Stoichiometries of all TRBP constructs for RNA length-dependent study. CD experiments were not performed for TRBP-dsRBD1 and TRBP-dsRBD2 in the presence of ds12 or ds16 due to their low binding affinities.
(TIF)

S2 Fig EMSA data for TRBP-dsRBD1 binding perfect duplex RNA. The radiograph images are presented, representing the increase of [TRBP-dsRBD1] from left to right. To the right of the gels are the Hill-style analyses of a set of two titrations. The experimental data (*black dots*) are averaged from the two independent experiments, with the black best-fit line produced from the determined K_d,app_ and n_H_ values, reported in Table 2.
(TIF)

S3 Fig EMSA data for TRBP-dsRBD2 binding perfect duplex RNA. The radiograph images are presented, representing the increase of [TRBP-dsRBD2] from left to right. To the right of the gels are the Hill-style analyses of a set of two titrations. The experimental data (*black dots*) are averaged from the two independent experiments, with the black best-fit line produced from the determined K_d,app_ and n_H_ values, reported in Table 2.
(TIF)

S4 Fig EMSA data for TRBP-ΔC binding perfect duplex RNA. The radiograph images are presented, representing the increase of [TRBP-ΔC] from left to right. To the right of the gels are the Hill-style analyses of a set of two titrations. The experimental data (*black dots*) are averaged from the two independent experiments, with the black best-fit line produced from the determined K_d,app_ and n_H_ values, reported in Table 2.
(TIF)

S5 Fig EMSA data for TRBP-dsRBD1 binding loop-dependent duplex RNA. The radiograph images are presented, representing the increase of [TRBP-dsRBD1] from left to right. To the right of the gels are the Hill-style analyses of a set of two titrations. The experimental data (*black dots*) are averaged from the two independent experiments, with the black best-fit line produced from the determined K_d,app_ and n_H_ values, reported in Table 2.
(TIF)

S6 Fig EMSA data for TRBP-dsRBD2 binding loop-dependent duplex RNA. The radiograph images are presented, representing the increase of [TRBP-dsRBD2] from left to right. To the right of the gels are the Hill-style analyses of a set of two titrations. The experimental data (*black dots*) are averaged from the two independent experiments, with the black best-fit line produced from the determined K_d,app_ and n_H_ values, reported in Table 2.
(TIF)

S7 Fig EMSA data for TRBP-ΔC binding loop-dependent duplex RNA. The radiograph images are presented, representing the increase of [TRBP-ΔC] from left to right. To the right of the gels are the Hill-style analyses of a set of two titrations. The experimental data (*black dots*) are averaged from the two independent experiments, with the black best-fit line produced from the determined K_d,app_ and n_H_ values, reported in Table 2.
(TIF)

S8 Fig EMSA data for TRBP-RBD1-Null and TRBP-RBD2-Null binding ds22. The radiograph images are presented, representing the increase of [TRBP-RBD1-Null] and [TRBP-RBD2-Null], respectively, from left to right. No binding is observed within the concentration window for these two constructs.
(TIF)
