# Supplementary material for: Helical Defects in MicroRNA Influence Protein Binding by TAR RNA Binding Protein
Source: PLoS One. 2015 Jan 21;10(1):e0116749. doi: 10.1371/journal.pone.0116749 (PMC4301919; doi:10.1371/journal.pone.0116749)
Supplement: S1 Table — (DOCX) [file pone.0116749.s001.docx]

| **Table S1.** Sequences of RNA oligonucleotides | | |
| --- | --- | --- |
| **RNA Construct** | Sequence (5´ to 3´) | ε (M^-1^ cm^-1^) |
| **12TS** | GUC AGC AGU GCC | 113,900 |
| **12BS** | GGC ACU GCU GAC | 110,300 |
| **16TS** | GUC AGC AGU GCC UUA G | 156,900 |
| **16BS** | C UAA GGC ACU GCU GAC | 151,300 |
| **22TS** | GUC AGC AGU GCC UUA GCA GCA C | 211,500 |
| **22BS** | G UGC UGC UAA GGC ACU GCU GAC | 206,300 |
| **33TS** | GGU CAG CAG UGC CUU AGC AGC ACG UAA AUA UGG | 326,800 |
| **33BS** | CCA UAU UUA CGU GCU GCU AAG GCA CUG CUG ACC | 319,300 |
| **44TS** | GGU CAG CAG UGC CUU AGC AGC ACG UAA AUA UUG GCG UUA AGA CC | 440,900 |
| **44BS** | GG UCU UAA CGC CAA UAU UUA CGU GCU GCU AAG GCA CUG CUG ACC | 422,900 |
| **ds16-tetra-stable** | GUC AGC AGU GCC UUA GUU CGC UAA GGC ACU GCU GAC | 344,200 |
| **ds16-**  **tetra-U** | GUC AGC AGU GCC UUA GUU UUC UAA GGC ACU GCU GAC | 351,400 |
| **ds16-**  **hexa-U** | GUC AGC AGU GCC UUA GUU UUU UCU AAG GCA CUG CUG AC | 370,800 |
| **ds16-**  **octa-U** | GUC AGC AGU GCC UUA GUU UUU UUU CUA AGG CAC UGC UGA C | 385,900 |
| **pre-mir-16-1** | UAG CAG CAC GUA AAU AUU GGC GUU AAG AUU CUA AAA UUA UCU CCA GUA UUA ACU GUG CUG CUG AA | 666,000 |
| **miR-16-1** | UAG CAG CAC GUA AAU AUU GGC G | 226,100 |
| **miR-16-1*** | CCA GUA UUA ACU GUG CUG CUG AA | 227,200 |
| **A-mis TS** | GGU CAG CAC GUA AAU AUU GUC C | 284,900 |
| **A-mis BS** | GGA CAG UAU UAA CGU GCU GUA C | 285,800 |
| **U-bulge TS** | GGU CAG CAC GUA AAU AUU GUC C | 284,900 |
| **U-bulge BS** | GGA CAG UAU UUA CUG UGC UGA UC | 290,400 |
