# Supplementary figures and images for: Helical Defects in MicroRNA Influence Protein Binding by TAR RNA Binding Protein
Source: PLoS One. 2015 Jan 21;10(1):e0116749. doi: 10.1371/journal.pone.0116749 (PMC4301919; doi:10.1371/journal.pone.0116749)

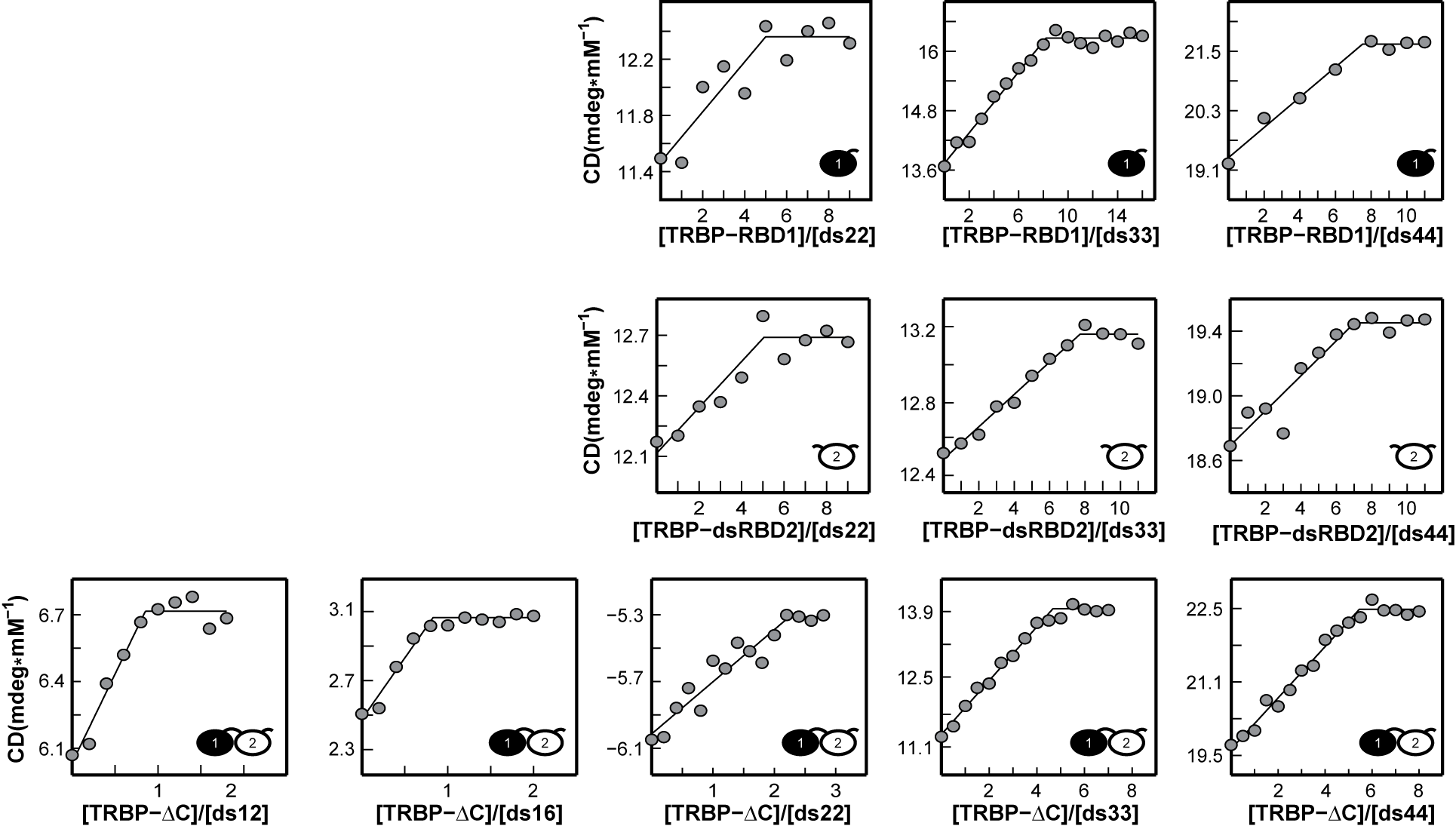

Supplement: S1 Fig — CD experiments were not performed for TRBP-dsRBD1 and TRBP-dsRBD2 in the presence of ds12 or ds16 due to their low binding affinities. (TIF) [file pone.0116749.s002.tif]

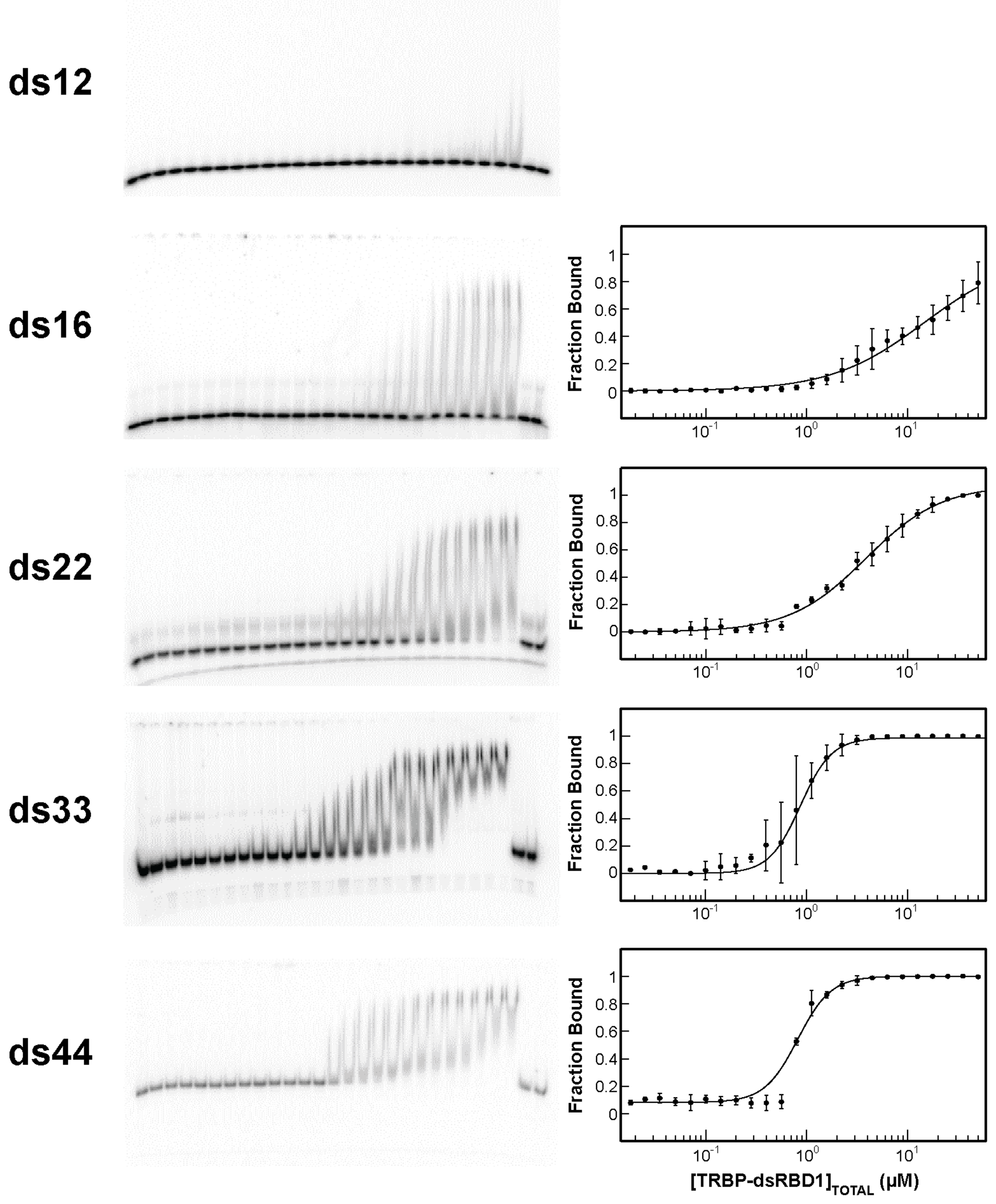

Supplement: S2 Fig — The radiograph images are presented, representing the increase of [TRBP-dsRBD1] from left to right. To the right of the gels are the Hill-style analyses of a set of two titrations. The experimental data (black dots) are averaged from the two independent experiments, with the black best-fit line produced from the determined Kd,app and nH values, reported in Table 2. (TIF) [file pone.0116749.s003.tif]

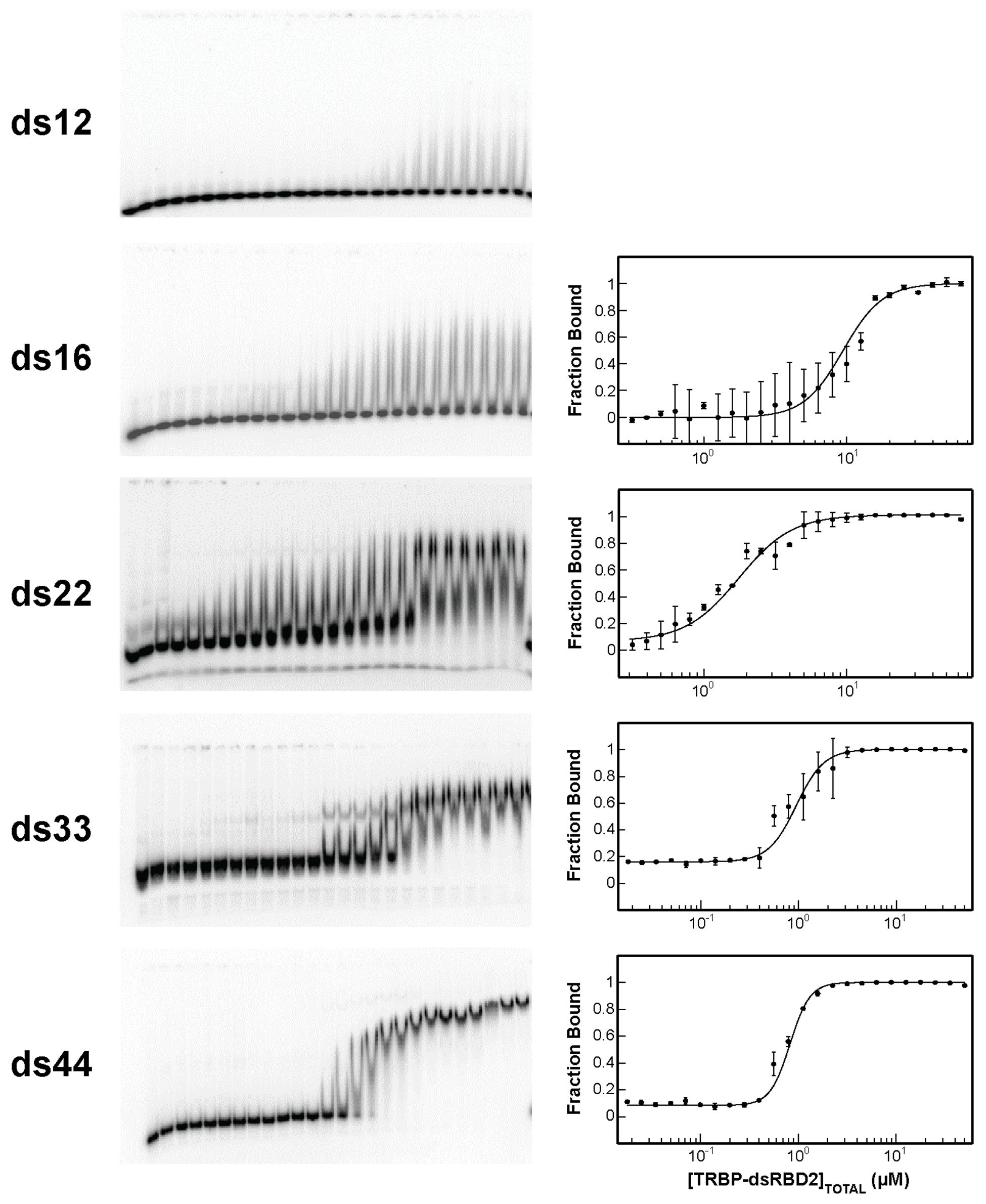

Supplement: S3 Fig — The radiograph images are presented, representing the increase of [TRBP-dsRBD2] from left to right. To the right of the gels are the Hill-style analyses of a set of two titrations. The experimental data (black dots) are averaged from the two independent experiments, with the black best-fit line produced from the determined Kd,app and nH values, reported in Table 2. (TIF) [file pone.0116749.s004.tif]

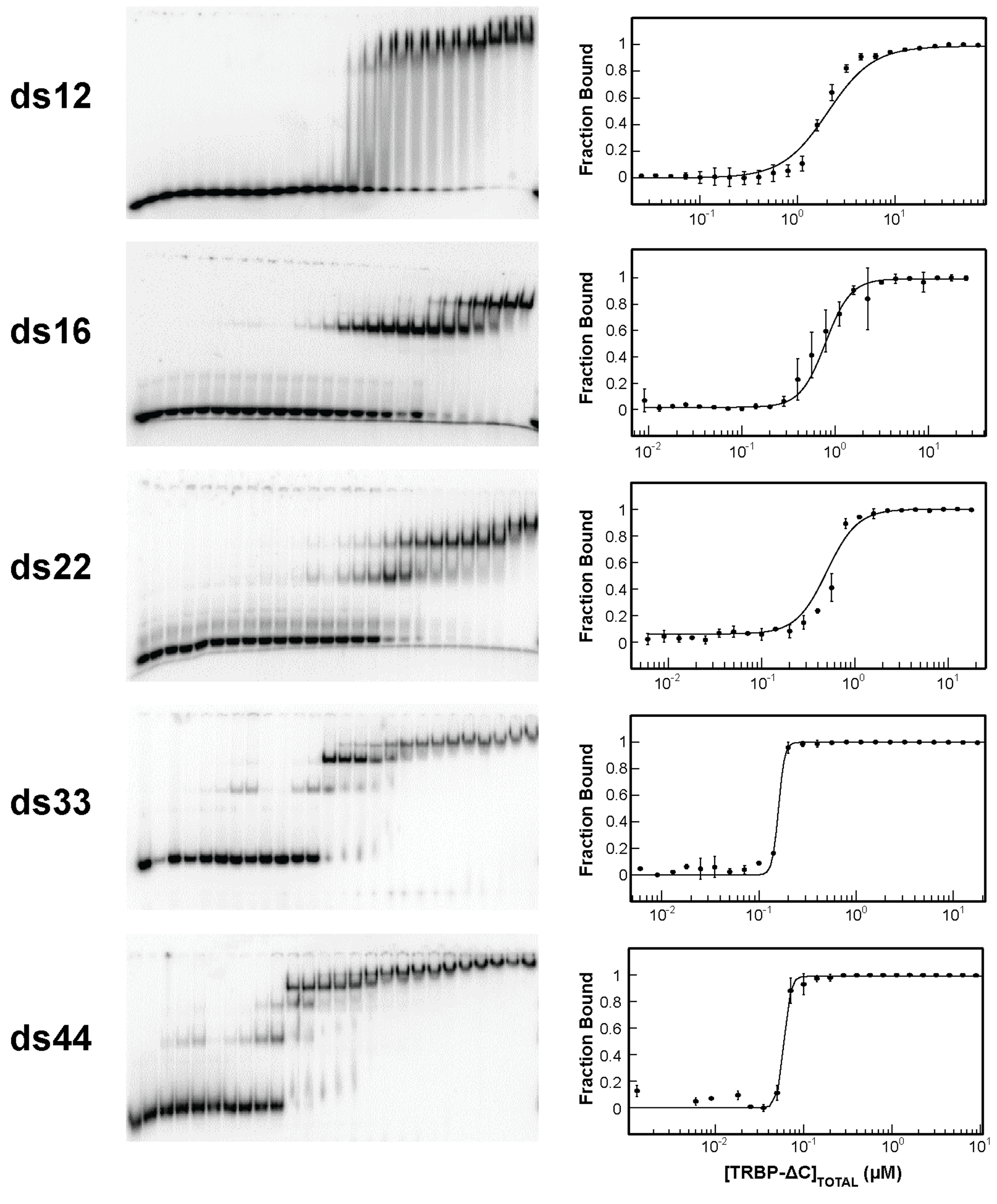

Supplement: S4 Fig — The radiograph images are presented, representing the increase of [TRBP-ΔC] from left to right. To the right of the gels are the Hill-style analyses of a set of two titrations. The experimental data (black dots) are averaged from the two independent experiments, with the black best-fit line produced from the determined Kd,app and nH values, reported in Table 2. (TIF) [file pone.0116749.s005.tif]

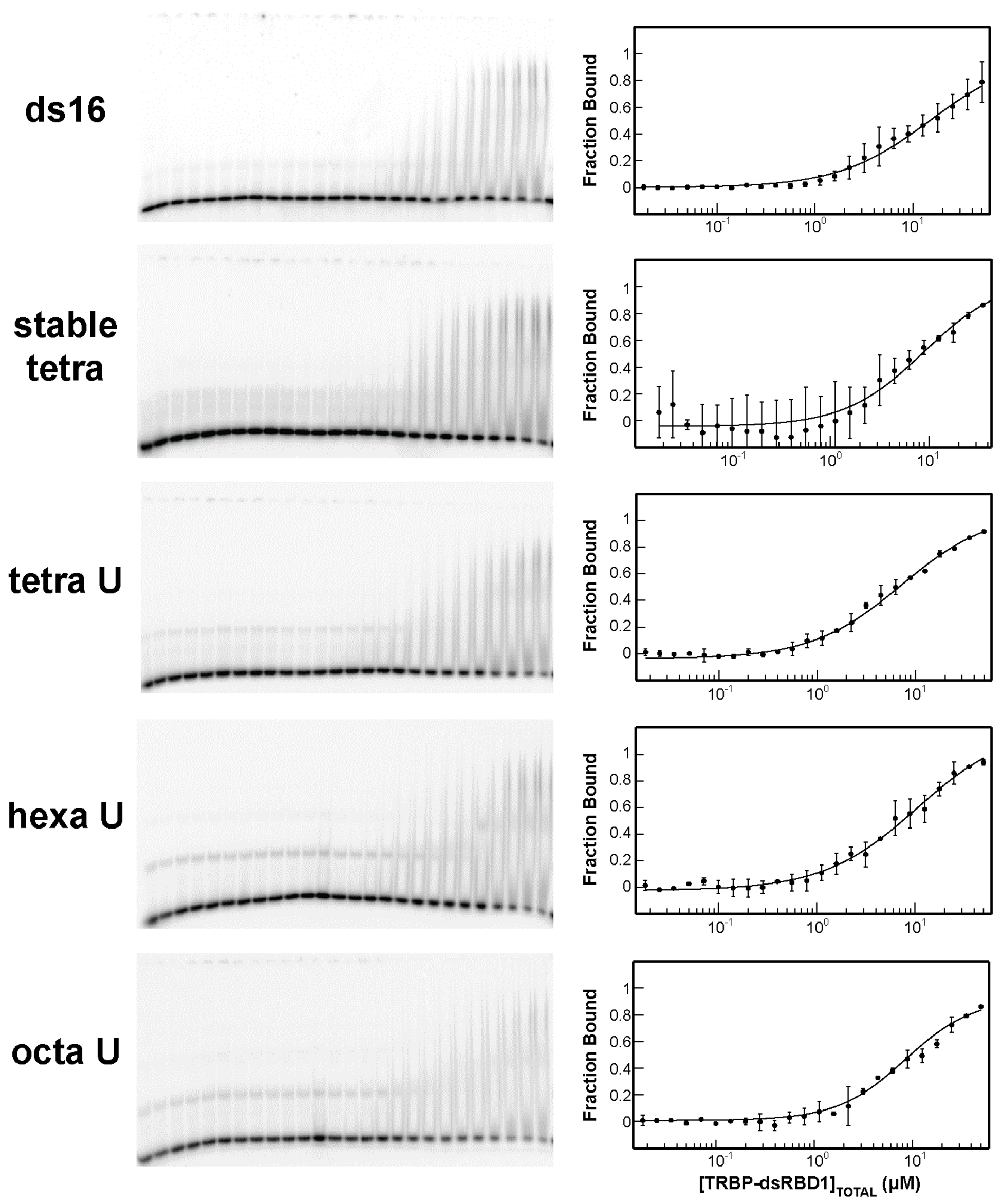

Supplement: S5 Fig — The radiograph images are presented, representing the increase of [TRBP-dsRBD1] from left to right. To the right of the gels are the Hill-style analyses of a set of two titrations. The experimental data (black dots) are averaged from the two independent experiments, with the black best-fit line produced from the determined Kd,app and nH values, reported in Table 2. (TIF) [file pone.0116749.s006.tif]

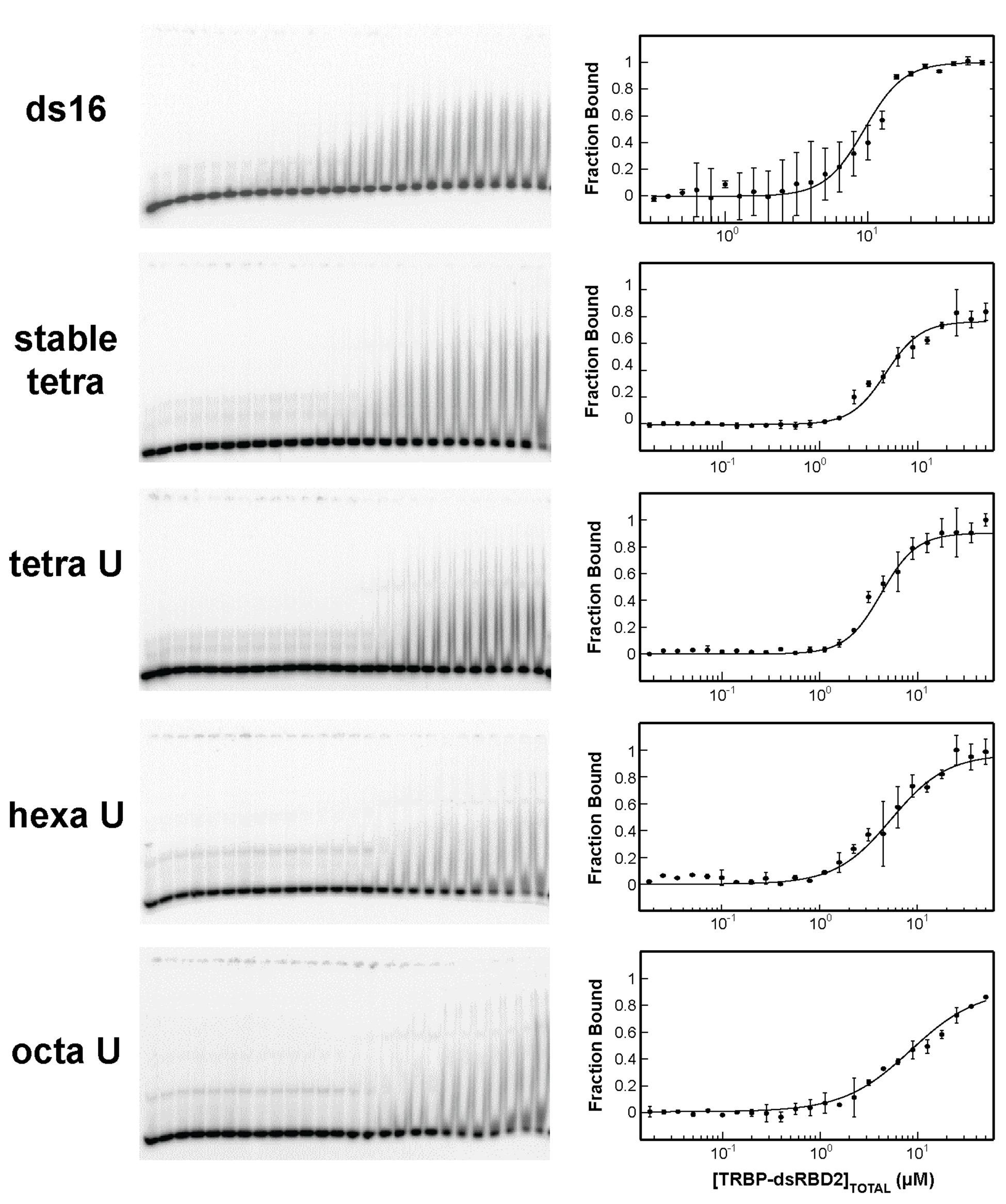

Supplement: S6 Fig — The radiograph images are presented, representing the increase of [TRBP-dsRBD2] from left to right. To the right of the gels are the Hill-style analyses of a set of two titrations. The experimental data (black dots) are averaged from the two independent experiments, with the black best-fit line produced from the determined Kd,app and nH values, reported in Table 2. (TIF) [file pone.0116749.s007.tif]

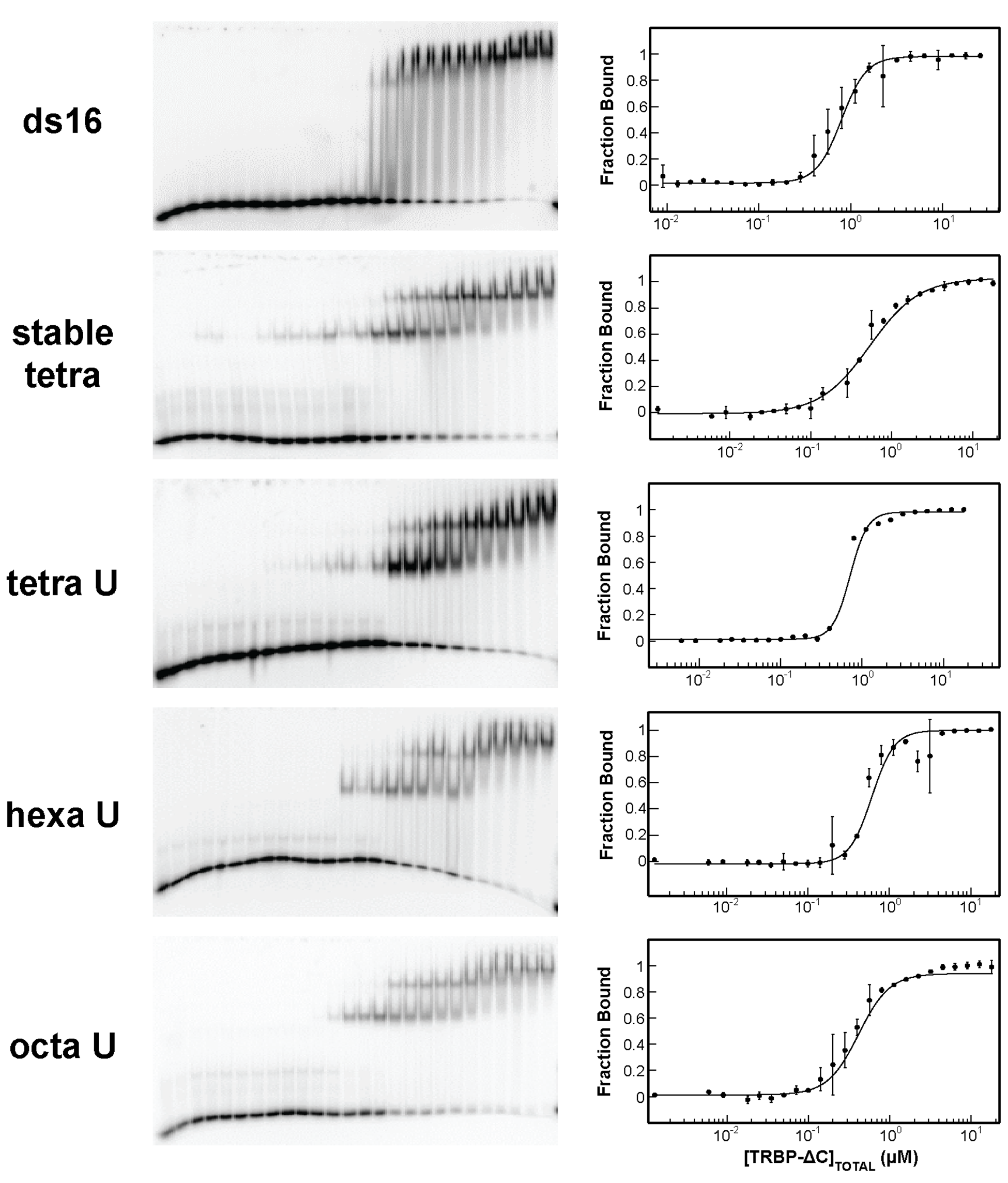

Supplement: S7 Fig — The radiograph images are presented, representing the increase of [TRBP-ΔC] from left to right. To the right of the gels are the Hill-style analyses of a set of two titrations. The experimental data (black dots) are averaged from the two independent experiments, with the black best-fit line produced from the determined Kd,app and nH values, reported in Table 2. (TIF) [file pone.0116749.s008.tif]

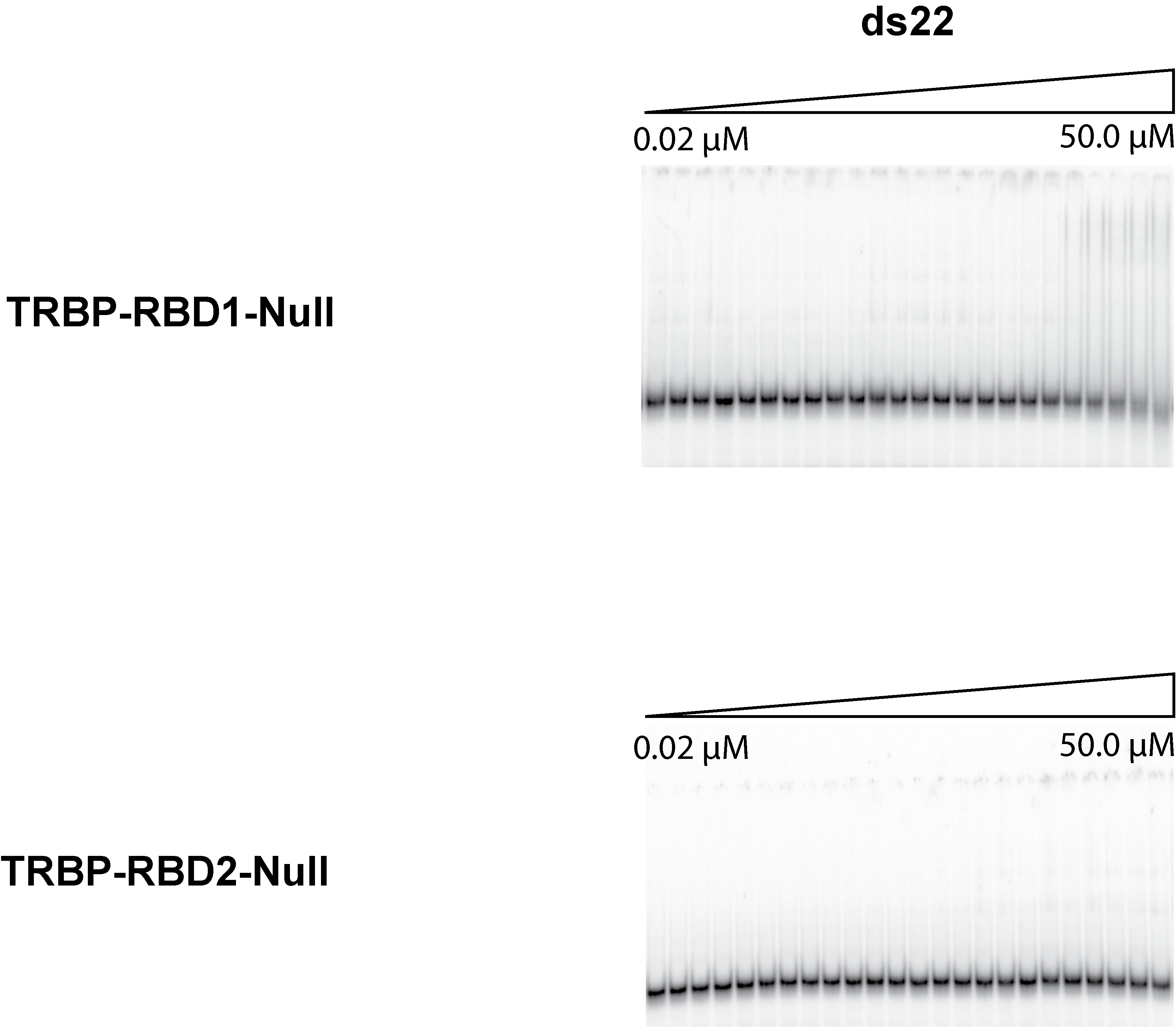

Supplement: S8 Fig — The radiograph images are presented, representing the increase of [TRBP-RBD1-Null] and [TRBP-RBD2-Null], respectively, from left to right. No binding is observed within the concentration window for these two constructs. (TIF) [file pone.0116749.s009.tif]
